# Interaction between photoperiod and variation in circadian rhythms in tomato

**DOI:** 10.1186/s12870-022-03565-1

**Published:** 2022-04-09

**Authors:** Yanli Xiang, Thomas Sapir, Pauline Rouillard, Marina Ferrand, José M. Jiménez-Gómez

**Affiliations:** 1 Université Paris-Saclay, INRAE, AgroParisTech, Institut Jean-Pierre Bourgin (IJPB), 78000 Versailles, France; 2 VIB-UGent Center for Plant Systems Biology, Technologiepark 71, 9052 Gent, Belgium; 3 Centro de Biotecnología y Genómica de Plantas, Universidad Politécnica de Madrid (UPM) - Instituto Nacional de Investigación y Tecnología Agraria y Alimentaria (INIA-CSIC), Madrid, Spain

**Keywords:** Tomato domestication, Circadian rhythms, Photoperiod, RNA-seq

## Abstract

**Background:**

Many biological processes follow circadian rhythmicity and are controlled by the circadian clock. Predictable environmental changes such as seasonal variation in photoperiod can modulate circadian rhythms, allowing organisms to adjust the timing of their biological processes to the time of the year. In some crops such as rice, barley or soybean, mutations in circadian clock genes have altered photoperiod sensitivity, enhancing their cultivability in specific seasons and latitudes. However, how changes in circadian rhythms interact with the perception of photoperiod in crops remain poorly studied. In tomato, the appearance during domestication of mutations in *EMPFINDLICHER IM DUNKELROTEN LICHT 1* (*EID1*, Solyc09g075080) and *NIGHT LIGHT-INDUCIBLE AND CLOCK-REGULATED GENE 2* (*LNK2*, Solyc01g068560) delayed both the phase and period of its circadian rhythms. The fact that variation in period and phase are separated in tomato provides an optimal tool to study how these factors affect the perception of photoperiod.

**Results:**

Here we develop tomato near isogenic lines carrying combinations of wild alleles of *EID1* and *LNK2* and show that they recreate the changes in phase and period that occurred during its domestication. We perform transcriptomic profiling of these near isogenic lines under two different photoperiods, and observe that EID1, but not LNK2, has a large effect on how the tomato transcriptome responds to photoperiod. This large effect of EID1 is likely a consequence of the global phase shift elicited by this gene in tomato's circadian rhythms.

**Conclusions:**

Our study shows that changes in phase that occurred during tomato domestication determine photoperiod perception in this species, while changes in period have little effect.

**Supplementary Information:**

The online version contains supplementary material available at 10.1186/s12870-022-03565-1.

## Background

Synchronization with the environment is crucial for survival. In order to efficiently anticipate predictable environmental changes linked to diurnal oscillations, all living organisms have developed endogenous timekeeping mechanisms named circadian clocks [1]. In plants, the circadian clock ensures the correct timing of crucial biological processes, such as growth, development, reproduction, photosynthesis or defense, just to name a few [2].

The circadian clock in plants is best studied in Arabidopsis, where it is organized in interlocked transcriptional feedback loops [3]. In this species, two important transcription factor families are at the core of the clock. The REVEILLE (RVE) family, a set of MYB-like proteins that includes CCA1/LHY, RVE6, RVE3 and RVE8 [4]; and the PSEUDORESPONSE REGULATOR (PRR) family, composed of PRR9, PRR7, PRR5, PRR3 and TOC1 that are sequentially expressed during the day [5]. Additional members of the circadian clock include proteins that enable environmental perception such as ZEITLUPE, GIGANTEA, EARLY FLOWERING 3, EARLY FLOWERING 4, LUX ARRHYTHMO or the NIGHT LIGHT-INDUCIBLE AND CLOCK-REGULATED family (LNKs) [6]. Most of these proteins are involved in light signaling and contribute to dynamically synchronize the circadian clock with the external cycles [7]. For example, these proteins reset the circadian clock each day by reading light and temperature signals at dawn or dusk [8]. Moreover, they can change the time of the day when some transcripts are expressed depending on the duration of the light period (photoperiod), allowing plants to time biological processes differently depending on the season [7, 9–13].

Because circadian rhythms can be modulated by the environment, their variation could be beneficial for adaptation to certain settings, and opens the possibility to selective pressures on the circadian clock. For example, plants that live near the equator where the duration of the day is constant along the year, could evolve different mechanisms to time their biological processes than plants at higher or lower latitudes that experience strong seasonal variation. Indeed, it has been found that plants adapted to higher latitudes naturally present longer free running circadian periods [14–16]. Moreover, allelic variations at circadian clock genes have been selected during crop domestication or breeding [17, 18]. In soybean, variation in circadian clock genes such as GI [19], PRR3 [20] or ELF3 [21] has been selected to control flowering time responses to photoperiod. The same has occurred in sugar beet with PRR3/7 [22], in barley with PPR3/7 [23] and ELF3 [24], and in pea and lentil with ELF3 [25].

Tomato is an interesting case because domestication significantly decelerated circadian rhythms through selection of knock-out alleles of two genes not identified as a source of variation in any other crop, *EID1* and *LNK2* [26, 27]. EID1 is an F-box protein first identified in tobacco as a clock and light regulated gene [28], that targets phytochrome A for degradation in Arabidopsis [29]. In tomato, a three base pair deletion in *EID1* appeared early during domestication causing a ~ 2 h phase delay of its circadian rhythms [26, 27]. LNK2 is part of a family of light inducible coactivators in Arabidopsis that interact with proteins of the RVE family to regulate the expression of clock genes [30–33]. In cultivated tomato, *LNK2* presents a large deletion that increases the period of its circadian rhythms [27]. The genomic regions of *EID1* and *LNK2* show signatures of positive selection and their mutations are fixed in cultivated tomatoes but do not exists in its closest wild ancestor *S. pimpinellifolium*, suggesting that phase and period changes have been beneficial for its domestication [26, 27]. Because both EID1 and LNK2 are involved in light signaling to the circadian clock, and tomato domestication started in the equatorial region of South America with constant photoperiods of ~ 12 h, it has been hypothesized that mutations in *EID1* and *LNK2* allowed tomato to expand its cultivation range to higher latitudes [26, 27].

Here we study the interaction between photoperiod and the mutations that delayed circadian rhythms in tomato. For this, we generate a set of tomato near isogenic lines that segregate for wild functional alleles of *EID1* and *LNK2*. We first confirm the validity of these lines and then study their transcriptional responses to variation in photoperiod and to the allelic configurations of *EID1* and *LNK2*. Our study contributes to understanding how variation in circadian rhythms affects the molecular state of tomato under different photoperiods.

## Results

### Generation and phenotyping of LNK2 + /EID1 + near isogenic lines

In order to investigate how mutations in EID1 and LNK2 interact with each other and how they affect the perception of photoperiod in tomato we generated near isogenic lines (NILs) that contain wild alleles of both genes in a cultivated tomato background. For this we performed several rounds of backcrossing in a set of previously developed NILs containing introgressions from the wild tomato *S. pimpinellifolium* (accession TO937) in a cultivated *S. lycopersicum* (accession Moneymaker) background [34]. We obtained a heterozygous line carrying reduced *S. pimpinellifolium* introgressions at the positions of *EID1* and *LNK2*. Whole genome short read sequencing of this line revealed the presence of 5 introgressions in chromosomes 1, 4, 5 (× 2) and 9 (Figure S1). The introgression at chromosome 1 comprised 125 genes and included the *LNK2* locus, while the introgression in chromosome 9 comprised 19 genes, including *EID1*. The progeny of this line was screened for homozygous individuals carrying exclusively the introgression in chromosome 1 (called line LNK2 +), the introgression in chromosome 9 (called line EID1 +) or both introgressions (EID1 + / LNK2 + , called line LE + hereafter). In addition, all lines selected carried an introgression at the bottom chromosome 4 that includes 208 genes and had been previously detected as a segregation distorter during the generation of the near isogenic line population [34].

**Fig. 1:**
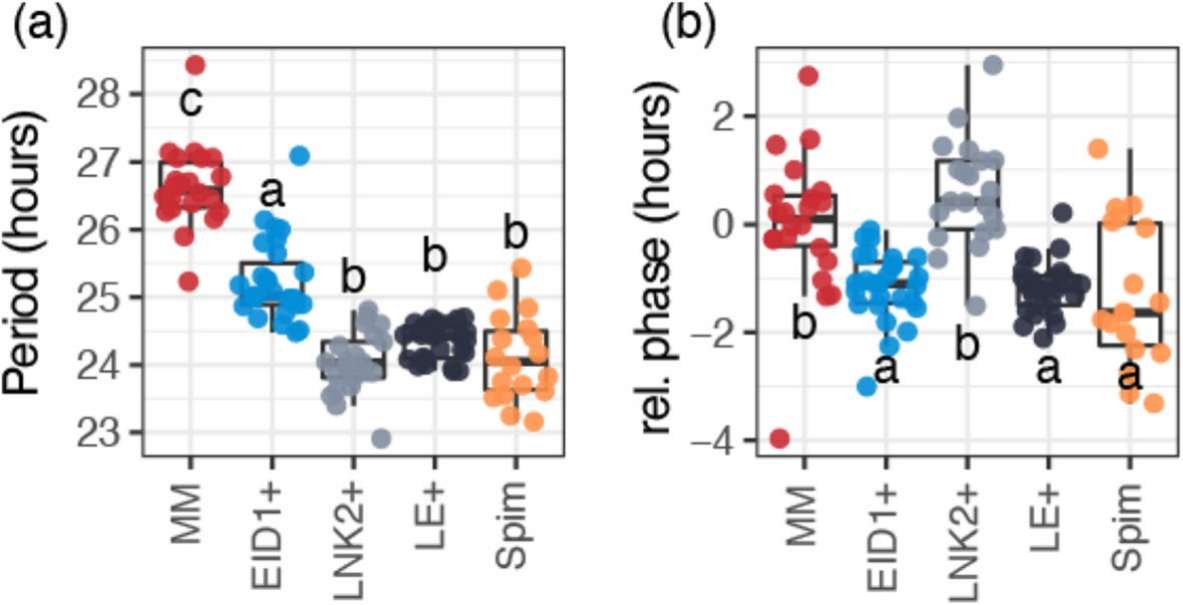
Circadian period **(a)** and relative phase **(b)** estimates in the near isogenic lines segregating for wild alleles of *EID1* and *LNK2*. Data comes from three independent experiments shown in Figure S2. Relative phase values were obtained by subtracting each phase value from the average of MM in each experiment. MM stands for *S. lycopersicum* cv. MoneyMaker. Spim stands for *S. pimpinellifolium.* Different letters in each boxplot indicate significant differences (*p* < 0.05, one-way ANOVA and Tukey’s post hoc HSD test)

We confirmed the functionality of the *S. pimpinellifolium* alleles of *LNK2* and *EID1* in these lines by characterizing its circadian rhythms in three independent experiments. As expected, wild *LNK2* alleles decreased the long circadian period observed in cultivated tomato but had no significant effect on the phase of leaf movements (Fig. 1). Wild *EID1* alleles reduced the circadian phase of cultivated tomato and also had a significant effect reducing its period, albeit not as strongly as *LNK2*. The combination of both wild alleles in the LE + line was sufficient to restore the period and phase to the values observed in the wild tomato *S. pimpinellifolium*, suggesting that the only mutations altering circadian rhythms in cultivated tomato are the ones present in *LNK2* and *EID1*. We did not observe consistent effects on the amplitude or quality (relative amplitude error) of circadian rhythms (Figure S2). In summary, the set of NILs generated recapitulates the variation in circadian rhythms observed between cultivated tomato and its closest wild relative *S. pimpinellifolium,* and allows studying the interaction between mutations in *EID1* and *LNK2*.

### Expression analyses

We performed RNA-seq on the near isogenic lines carrying wild alleles of *EID1* and *LNK2* to determine their role in photoperiod perception in tomato. For this, we grew the lines under long day (LD) and short day (SD) conditions, and we collected leaf samples from three biological replicates two hours after lights on (Zeitgeber time 2, ZT2). This time of the day was chosen to coincide with the highest expression of *EID1* (ZT0) and *LNK2* (ZT4) in a published time course experiment in tomato [26] (Figure S3).

We obtained an average of 34.8 million read pairs per sample (minimum of 19.8 million and maximum of 43.8), that were aligned to the tomato reference genome sequence with an average success rate of 92.9%. Principal component analysis (Figure S4) revealed photoperiod as the mayor factor controlling the variance observed among samples (associated to principal component 1, PC1, 65%), while other factors that might be related to the genotype at *LNK2* and *EID1* had smaller effect (associated to PC2, 19%).

### Variation in response to genotype

**Fig. 2:**
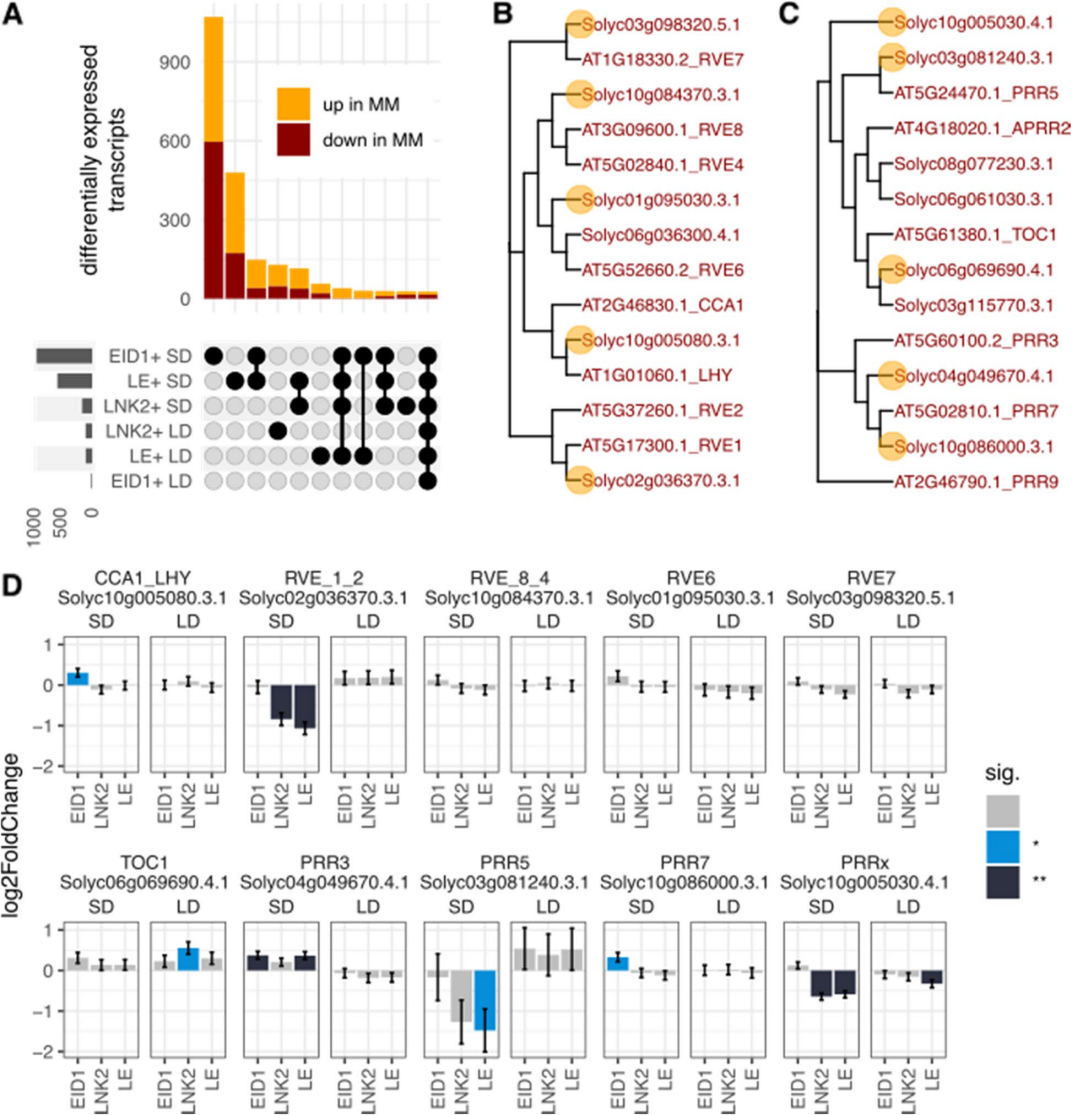
Differentially expressed genes in response to wild alleles of *LNK2* and *EID1* in each photoperiod separately. **A** Horizontal bars represent the number of differentially expressed transcripts in each genotype and condition. Vertical bars represent the number of differentially expressed genes in each set, with a set being a combination of comparisons. Only sets with more than 20 genes are shown. **B** and **C** Phylogenetic tree with protein sequences from Arabidopsis and tomato for the RVE family (**B**) and the PRR family (**C**) of circadian clock genes. Arabidopsis and tomato protein names are highlighted in gray and red respectively. Tomato proteins whose transcripts oscillate during the diel cycle are marked with an orange dot. **D** Log2 fold change in expression (± standard error) between *S. lycopersicum* var MoneyMaker (MM) and each of the near isogenic lines in this work. SD and LD stand for short days and long days. Color of the bars indicate the significance of each log2 fold change, with non-significant values in gray, q < 0.05 in blue and q < 0.01 in black

We first investigated if wild alleles of *LNK2* and *EID1* affect gene expression under specific photoperiod by comparing each NIL to *S. lycopersicum* cv Moneymaker (MM) in each photoperiod separately. Figure 2A presents horizontal bars with the number of differentially expressed transcripts in each of these comparisons. We found a total of 2316 differentially expressed transcripts (9.95% of the analyzed transcriptome), with the majority of them (90.75%) showing differences in short days, and only 18.86% showing differences in long days. The largest set of differentially expressed transcripts was found between the EID1 + line and MM in short days (1426 transcripts), with the majority of those being exclusive to this comparison (1071 transcripts, represented by the first vertical bar in Fig. 2A). The second comparison with most abundant differentially expressed transcripts was between the LE + line and MM in short days (934 transcripts, 480 unique to this comparison, represented by the second vertical bar in Fig. 2A). Interestingly, only 56 transcripts were differentially expressed between the EID1 + line and MM in LD, indicating that the large effect of EID1 on expression is photoperiod specific. Transcripts affected by EID1 + in short days were enriched in GO terms related to biological processes such as DNA replication, microtubule movement or photosynthesis (Table S2).

In contrast to the large effect found for EID1, the addition of wild alleles of *LNK2* affected only 297 transcripts in short days and 254 in long days, suggesting that EID1 has a more important role shaping gene expression than LNK2. GO terms of biological processes associated to the transcripts affected by LNK2 are translation or photosynthesis (Table S2). We were surprised by the mild effect of LNK2 + on expression, since LNK proteins in Arabidopsis are involved in transcriptional initiation of clock genes in the PSEUDO RESPONSE REGULATOR (PRR) family such as PRR5 and TOC1, and affects the expression of members of the REVEILLE (RVE) family such as CCA1 or LHY [30–33, 35]. To investigate whether this is the case in tomato, we identified the homologs of the PRR and RVE families from Arabidopsis (Fig. 2, panels B and C). While genes such as *RVE7*, *PRR5* or *PRR7* had clear homologs in tomato, some others had been lost (*CCA1* / *LHY*) or had undergone duplication (*RVE6* and *TOC1*). To increase the likelihood of functional homology we considered as homologs only tomato transcripts whose expression oscillated during the diel cycle in a published RNA-seq time-course experiment [26].

Among the *RVE* genes in tomato, wild alleles of *LNK2* significantly decreased the expression of the homolog of *RVE1* and *RVE2* in short days, but had no effect on the homolog of *CCA1* and *LHY*, as it does in Arabidopsis (Fig. 2). Interestingly, the homolog of *CCA1* and *LHY* in tomato was affected by the addition of wild alleles of *EID1* only in short days. Among the PRRs in tomato, wild alleles of *LNK2* significantly decreased the expression of a *PRRx* transcript for which we could not conclude a closest homolog in Arabidopsis. In concordance with what was observed in Arabidopsis, *LNK2* alleles affected the expression of the homolog of *TOC1*, although this effect was only significant at the 0.05 level in long days, and only in the absence of functional alleles of *EID1*. In addition, wild alleles of *LNK2* strongly decreased the expression of the homolog of *PRR5* in short days, albeit not significantly due to large variation among samples (*p* = 0.16 in LNK2 + and *p* = 0.049 in *LE* +). Finally, wild alleles of *EID1* increased the expression of *PRR3* and *PRR7* in short days. Interestingly, most of the significant differences of expression observed were photoperiod-specific, suggesting a complex seasonal effect in the regulatory function of LNK2 and EID1. In summary, although some transcripts in the PRR and RVE families in tomato are affected by allelic variation in *LNK2* and *EID1*, we cannot conclude that the mechanism of action of their proteins in tomato are similar to those previously defined in Arabidopsis.

### Variation in response to photoperiod

**Fig. 3:**
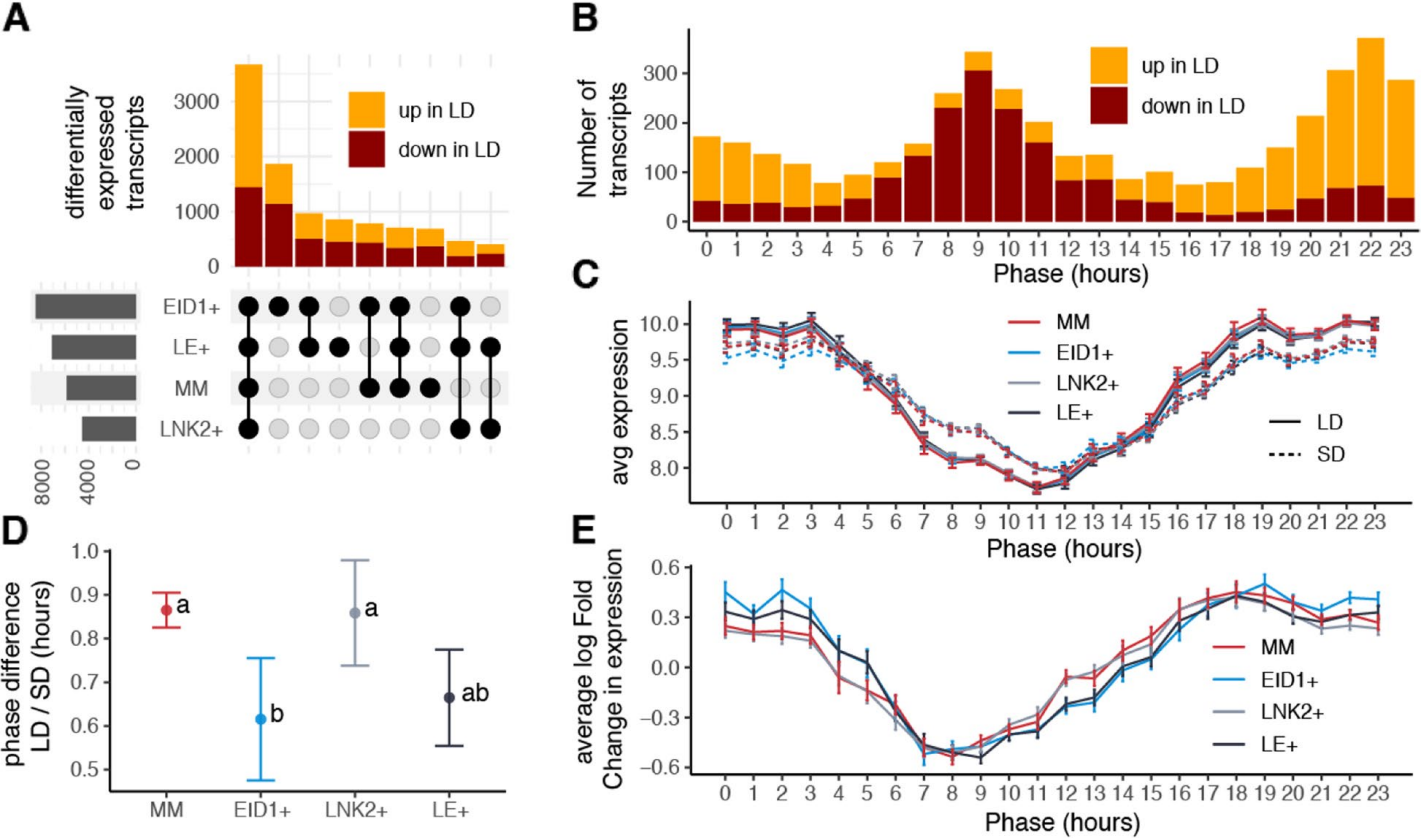
**A** Differentially expressed transcripts in response to photoperiod in each genotype separately. Horizontal bars represent the total number of differentially expressed transcripts in each genotype. Vertical bars represent the number of differentially expressed genes in each set, with a set being a combination of comparisons. **B** Number of cycling transcripts whose expression is affected by changes in photoperiod grouped by their phase. **C** Average expression of transcripts whose expression oscillated during the day grouped by their phase, genotype and condition. **D** Differences between the estimated phase in long days and short days. Error bars represent the standard deviation of the mean. Different letters in each dot indicate significant differences (*p* < 0.05, using two-way ANOVA and estimated marginal means). **E** Average log2 fold change in expression induced by photoperiod in cycling genes grouped by genotype and phase of expression. Error bars represent the standard error of the mean

To further explore the interaction of *LNK2* and *EID1* alleles with photoperiod in tomato we analyzed photoperiod sensitivity in each NIL. Fifty percent of the characterized transcriptome (11,475 transcripts out of 23,269) was significantly affected by photoperiod in at least one genotype (Fig. 3). We found roughly the same number of genes upregulated and downregulated by the long day treatment (50.22% upregulated in long days). Moreover, when a gene was differentially expressed in more than one genotype, the direction of the differential expression was consistent between genotypes in 99.5% of the cases.

We divided transcripts significantly responding to photoperiod in groups according to their occurrence in the different genotypes (Fig. 3A). Horizontal bars in Fig. 3A represent the number of differentially expressed transcripts between long days and short days in each genotype separately, which was higher in lines carrying wild alleles of *EID1* (EID1 + and LE +). The largest set is formed by 3671 transcripts responding to photoperiod in all genotypes, followed by 1866 transcripts affected exclusively in the EID1 + genotype, 978 transcripts deregulated simultaneously in LE + and EID1 + , and 855 differentially expressed in LE + (Fig. 3A). These results suggest that, as observed before and despite a prevalent effect of photoperiod independently of the genotype, the mutation at the *EID1* locus is associated with stronger changes in gene expression, thus stronger photoperiod sensitivity, than the *LNK2* mutation. GO terms overrepresented among transcripts responding to photoperiod in all lines were associated to translation or cell redox homeostasis, while those among transcripts responding to photoperiod only in EID1 + lines were associated to translation and photosynthesis (Table S2).

Previous studies have determined that changes in photoperiod can impact the expression pattern of transcripts whose expression cycles throughout the day [9–11]. We obtained a list of such transcripts in tomato together with their phase (the time of the day when they show their maximum expression) using previously published RNA-seq data from seedlings from cultivated tomato and its wild relative *S. pennellii* sampled every 4 h for one day in 12 h light / 12 h dark photoperiods [26] (Table S3). As observed in other plants, the phases of cycling genes in tomato were not evenly distributed throughout the day, with a majority of transcripts having phases right before dawn (ZT0) or dusk (ZT12) (Figure S5a) [9, 10]. Changes in photoperiod significantly affected the expression of more than two thirds of cycling transcripts (4181 out of 6017) whose phases were equally distributed all along the diel cycle (Figure S5b), indicating that changes in photoperiod affect the expression of genes expressed at all times of the day. However, while the majority of cycling genes whose phase occurred at the end of the day were downregulated in long days, cycling genes with phases at the end of the night were mostly upregulated (Fig. 3B). Such a marked time of the day specific effect of photoperiod could result from a shift in the phase of circadian rhythms between conditions.

In order to explore this possibility, we evaluated our data using the molecular timetable method that allows phase estimation using a single time point [36, 37]. The method infers the internal phase of the sample by evaluating the relative expression of "time-indicating" cycling transcripts with known maximum time of expression. Thus, in samples taken at ZT2, we would expect higher expression of transcripts whose expression normally peak in the late night and early morning and lower on transcripts whose expression peak later in the day, which is what we observed in our experiment (Fig. 3C). Expression differences between samples in long days and samples in short days were consistent with the results observed before, with long days inducing on average higher expression in dusk genes and lower expression in dawn genes. These results indicate an advanced phase of the circadian clock of tomato under short days compared to long days, as reported for other species before [9–11]. In summary, photoperiod shifts the timing of expression of cycling genes in tomato, thus causing substantial environment specific transcriptional differences.

In order to investigate if wild alleles of *EID1* or *LNK2* affect photoperiodic responses, we estimated the phase differences between the molecular timetable curves in each of our near isogenic lines. Plants in long days have an average of 0.8 h phase delay with respect to plants grown in short days (Fig. 3D). Importantly, phase differences caused by photoperiod were smaller when wild alleles of *EID1* were present, again suggesting that variation in *EID1* but not *LNK2* modulates the phase of cycling genes. We looked for additional confirmation of the effect of photoperiod and *EID1* on the phase of cycling genes by measuring the magnitude of expression changes at each phase bin, since genes with different phases are expected to present larger expression changes at specific times of the day. In Fig. 3C we see that the change of phase between long days and short days translates in low expression differences caused by photoperiod around ZT4 and ZT14 and strong differences around ZT8 and ZT20. Representation of the average fold change in expression between photoperiods in each genotype shows that indeed this is the case, with log fold change values at ZT4 and ZT14 approaching zero (Fig. 3E). Moreover, log fold change differences also separate lines carrying different alleles of *EID1* in two groups that present different profiles (Fig. 3E). In summary, our results show that photoperiod has a strong effect on the phase of expression of tomato transcripts, and that variation in the *EID1* locus, but not in *LNK2*, modulate this effect.

## Conclusions

Here we developed a set of isogenic cultivated tomato lines segregating for wild, functional alleles of *LNK2* and *EID1*, the two genes responsible for delayed circadian rhythms in cultivated tomato [26, 27]. Although near isogenic lines containing wild alleles of *EID1* and *LNK2* were developed in our previous works [26, 27], the current lines represent a great improvement over those as a genetic tool to study the role of these genes in tomato physiology. First, the previously existing lines contained large introgressed fragments involving hundreds, if not thousands of genes from the wild species, while the lines developed in this work contain wild alleles in only 125 genes in the region of *LNK2* and 19 genes in the region of *EID1* (Figure S1). It is worth noting that these lines also contain 208 genes with wild alleles at the bottom of chromosome 4, in a region that caused a strong segregation distortion when the population was generated [34]. Despite all our efforts backcrossing these lines, we did not manage to remove this region, although its presence in all lines ensures that it cannot be responsible for the observed differences between lines. Second, the wild donor that we chose to develop the lines in this work is *S. pimpinellifolium*, instead of *S. pennellii* in the previous lines. While *S. pimpinellifolium* is the closest wild relative of cultivated tomato, *S. pennellii* is one of the most distant wild relatives. The closer genetic relationship between donor and acceptor is likely to induce less unintended effects of additional genes present in the introgressions, since they should contain fewer sequence polymorphisms between cultivated and wild alleles.

We confirmed the functionality of the *S. pimpinellifolium* alleles of *EID1* and *LNK2* in the isogenic lines by characterizing their circadian leaf movements (Fig. 1). The results coincide with previous work showing that wild alleles of *LNK2* and *EID1* respectively revert the period and phase delay observed in cultivated tomato [26, 27]. It is interesting to observe in all three independent experiments showed an additional albeit secondary effect of *EID1* in period (Fig. 1). The effect of *EID1* in period coincides in its direction with the shortening of period caused by *LNK2*, confirming that both genes act synergistically and reinforcing the hypothesis of positive selection towards delayed circadian rhythms in cultivated tomato.

The development of these isogenic lines allowed us for the first time to study the interaction of the different alleles of *EID1* and *LNK2*, since no other line was developed before where wild alleles of both genes were present in a cultivated tomato background. The phenotype of the LE + line resembles perfectly that of the tomato wild relative *S. pimpinellifolium* (Figure S2). This confirms the results of our previously published QTL analysis that identified only two QTLs controlling this trait in a RIL population between cultivated tomato and *S. pimpinellifolium*, predicting that the mutations in *LNK2* and *EID1* are sufficient to generate the delayed circadian rhythm phenotype of cultivated tomato.

We studied the transcriptional changes caused by variation in photoperiod and in the different alleles of *EID1* and *LNK2* at the time when their transcripts are most abundant. Among the two genes studied, *EID1* affected the transcription of many more genes than *LNK2*. The smaller effect of *LNK2* could be explained by redundancy of its protein with other members of its family, that both in Arabidopsis and tomato is composed of 4 members (Figure S6). In Arabidopsis the *lnk2* mutant also shows limited phenotypic differences with the wild type plant as compared with the *lnk1;lnk2* double mutant [31] or the *lnk1;lnk2;lnk3;lnk4* quadruple mutant [35]. In addition, it is also possible that EID1 has a more general role shaping plant expression because of its role regulating photoreceptors [29, 38], therefore controlling the extensive light signaling network in plants, while LNK2 acts more specifically within the circadian clock and anthocyanin biosynthesis pathways [32, 35]. Nevertheless, we specifically looked for expression variation among the homologs of circadian clock genes in tomato, and did not observe strong differences. One possible explanation for this is that our samples were collected at ZT2, and the changes in expression triggered by *LNK2* are observed later in the day [30, 32, 33].

The transcriptional response to photoperiod was much larger than the response elicited by the genotype at the *EID1* or *LNK2* loci, affecting roughly half of the tomato transcriptome. This strong response is difficult to compare with previous studies that used other species, methods, significance thresholds and sample collection times. However, scanning of the literature revealed photoperiod effects that range from 50% of the transcriptome in the perennial grass *Panicum hallii* [9], to 20% in Medicago or Arabidopsis [39].

The large number of transcripts affected by photoperiod in our experiment is likely to be caused, at least in part, by the phase change induced by the light treatment. In effect, most transcripts whose expression oscillate during the day are significantly affected by photoperiod following a pattern in which afternoon-expressed genes are downregulated and late-night-expressed genes are upregulated. Using the molecular timetable method we found that this pattern would fit a global phase delay of cycling genes in response to long days, which is similar to what has been observed in other plants such as Arabidopsis [10, 11] and *Panicum hallii* [9]. In contrast, while our method based on a single time point estimates an average phase delay of 0.8 h (Fig. 3D), full time-course datasets performed in Arabidopsis and *Panicum hallii* [9, 10] estimate this phase change to be around 4 h. It is very likely that this discordance in the estimation of phase differences induced by photoperiod between our study and those of others is due to the low accuracy of our method, which is based in a prediction from a single time point, while the other studies are based on datasets with multiple samples collected at regular intervals during one or various days [9, 10]. Phase estimates using full time-course data are more precise due to simpler calculations, but methods that estimate phase from a single time point are gaining popularity because of their low cost and experimental simplification [36, 40, 41]. Nevertheless, precise photoperiod-induced phase change estimation in tomato would require measuring expression from samples collected at regular intervals during at least one-day in both conditions.

In summary, our work reveals a role of EID1 but not LNK2 in the perception of photoperiod in tomato through modulation of the phase of expression of cycling genes. Changes in photoperiod sensibility could have been important for tomato to better adapt to variation in day length in latitudes outside the tropics.

## Methods

### Plant material

Tomato near isogenic lines LNK2 + , EID1 + and LNK + /EID1 + (hereafter called LE +) were generated from a set of introgression lines derived from a cross between *S. lycopersicum* cv Moneymaker and *S. pimpinellifolium* accession TO-937, kindly provided by A. Monforte [34, 42]. Introgression lines SP1-2 and SP9-2 from this population, containing *S. pimpinellifolium* alleles in the region of *LNK2* and *EID1*, were backcrossed to the cultivated parent Moneymaker. Recombinants that reduced the introgressed regions were selected for two consecutive generations using markers detailed in Table S1, screening a total of 264 plants per line. Independent lines with reduced introgressions containing *S. pimpinellifolium* alleles of *EID1* and *LNK2* were crossed, and a whole genome sequencing was performed in a single F1 individual. Genotyping of short reads revealed the presence of introgressions in chromosomes 1 (the region of *LNK2*), 4, 5 (two introgressions) and 9 (the region of *EID1*) (Figure S1). The segregating progeny of this F1 line was genotyped for all these regions using markers detailed in Table S1 to select lines that had only the introgression in chromosome 1 containing *LNK2* (LNK2 +), only the introgression in chromosome 9 containing *EID1* (EID1 +) or introgressions only in chromosomes 1 and 9 (LNK2 + /EID1 + , hereafter LE +). In addition, all plants screened contained *S. pimpinellifolium* alleles on the distal region of chromosome 4 due to the presence of a segregation distortion locus identified during the generation of the population [34, 42].

### Genotyping tomato lines

A single F1 individual resulting from the cross of two lines carrying reduced *S. pimpinellifolium* introgressions in the region of *LNK2* and *EID1* (see above) was sequenced for whole genome genotyping. To do this, DNA from leaf tissue was extracted using the DNeasy Plant extraction kit from Qiagen following manufacturer's instructions. A single sequencing library was constructed using the standard Illumina method, and sequenced in an Illumina NovaSeq 6000 system, yielding 361,774,304 pairs of 150 bp reads. In order to assign alleles in the F1 hybrid to cultivated and wild tomato we obtained publicly available short reads from *S. pimpinellifolium* accession LA1589 and *S. lycopersicum* cv. Moneymaker [43, 44]. Reads from all three genotypes were independently aligned to the tomato reference genome v2.5 using Bowtie2 version 2.3.4.2 with default parameters [45]. Previous to variant calling, reads with mapping quality lower than 5 were discarded using samtools v1.7 [46], duplicated reads were removed using Picardtools (http://broadinstitute.github.io/picard) and indels were realigned using GATK IndelRealigner [47]. We called variants in all three alignments simultaneously using GATK UnifiedGenotyper [47]. Presence/absence of *S. pimpinellifolium* alleles in the F1 hybrid was scored at 3,396,011 biallelic positions where Moneymaker and LA1589 were homozygous and different from each other, and the hybrid was heterozygous.

### Leaf movement essays

Three different experiments to measure leaf movements were conducted following a protocol that has been described in detail elsewhere [48]. Briefly, seedlings were entrained in an environmental chamber to 12 h light / 12 h dark and 24 °C for seven to ten days. On the last day of entrainment, a polystyrene ball was attached to the tip of one cotyledon of each seedling and the conditions changed to constant light and temperature. Images of the seedlings were taken each 30 min for five days in constant conditions using Pentax Optio WG-1 digital cameras. Image analysis to extract the vertical position of the polystyrene ball was performed using ImageJ [48, 49]. Estimates for circadian variables were obtained via fast Fourier transform nonlinear least-squares analysis using BioDare2 (biodare2.ed.ac.uk) [50]. Only seedlings with error (ERR) values below 0.4 were considered for statistical analyses. Phase values were obtained with the "phase by fit" method that reports the phase of best fitting cosine wave with a period matching the estimated period of the sample. Phase values are ZT values, as they are divided by the estimated period for the sample.

### RNA-seq analysis

RNA-seq was conducted in two consecutive experiments in the same controlled environment chamber set first to LD (16 h light / 8 h dark, 24C) and then to SD (8 h light / 16 h light, 24 C). We collected leaf samples from 14-day old plants 2 h after dawn (ZT2), coinciding with the maximum peak of expression of *EID1* and *LNK2*. Three biological replicates were collected from each genotype and condition, and total RNA was extracted with the RNeasy Plant Mini Kit from Qiagen. Libraries were prepared according to the Illumina TruSeq RNA protocol and sequenced in two lanes of a Novaseq 6000 system, yielding 834,954,102 150 bp read pairs (per sample average of 34,789,754, minimum of 19,797,369).

Reads for each biological replicate were aligned independently to the tomato genome reference sequence v4.0 using hisat2 v2.1.0 [51], allowing a maximum intron length of 115400 bp (the largest intron in the tomato genome annotation ITAG4.1). An average of 92.9% of the reads aligned to the reference (minimum of 89.8%). The number of reads per transcript in the ITAG4.1 annotation was counted with the featureCounts function in the Rsubread R package with default parameters [52]. Only transcripts that presented more than 10 reads in all samples together were used in downstream analysis, leaving us with 23,226 transcripts out of the 34,688 transcripts present in the annotation. Sample homogeneity was surveyed the with the plotPCA function in the DEseq2 package in R [53]. Differential expression between each genotype and condition was calculated with DEseq2 using two different models. The first model aimed to obtain lists of differentially expressed transcripts between each genotype and *S. lycopersicum* cv Moneymaker in each photoperiod separately, and contained a unique variable with 8 factors grouping the genotype and condition for each sample ("MM_LD", "MM_SD","LNK2_LD", "LNK2_SD", "EID1_LD", "EID1_SD", "LE_LD" and "LE_SD"). The second model was conceived to test the effect of photoperiod in each genotype and included the three variables "photoperiod", "genotype at EID1" and "genotype at LNK2", as well as all its possible interactions (photoperiod + EID1 + LNK2 + photoperiod:EID1 + photoperiod:LNK2 + EID1:LNK2 + photoperiod:EID1:LNK2). From this model we extracted the effect of photoperiod in each genotype. In both models we considered as differentially expressed those transcripts with a q-value lower than 0.01. A dataset with normalized values for each sample used in all downstream analyses and graphical representations was generated using the vst function in the DEseq2 R package [53].

For functional analysis of differentially expressed genes, GO terms in the ITAG4.1 genome annotation were assigned to the 23,226 transcripts that presented more than 10 reads in all samples. Overrepresentation analysis was performed with the GOseq R package based on the Wallenius non-central hyper-geometric distribution [54]. GO categories with q values smaller than 0.05 (adjusted using the Benjamini and Hochberg's method) were considered as significantly overrepresented.

### Homologs of the PSEUDO-RESPONSE REGULATOR and REVEILLE gene families in tomato

To obtain the homologs of PRRs and RVE genes in tomato, we compared the protein sequences from the following Arabidopsis TAIR10 ids: AT1G01060.1 (LHY), AT1G18330.2 (RVE7), AT2G46830.1 (CCA1), AT3G09600.1, (RVE8), AT5G02840.1 (RVE4), AT5G17300.1 (RVE1), AT5G37260.1 (RVE2) AT5G52660.2 (RVE6), AT2G46790.1 (PRR9), AT4G18020.1 (APRR2), AT5G02810.1 (PRR7), AT5G24470.1 (PRR5), AT5G60100.2 (PRR3) and AT5G61380.1 (TOC1) onto the tomato protein sequences from annotation ITAG4.1 using standalone BLAST + [55]. For the RVE family, we selected all matching tomato proteins with a bit score greater than 90, and for the PRR family, we selected those with a p value lower than 1e-40. Only tomato transcripts whose expression showed oscillation during the diel cycle were considered homologs. Retrieval and management of sequences was performed with the seqinr package in R [17] and neighbor-joining phylogenetic trees were constructed and drawn with the ape package [56] and the ggtree package [57] in R respectively.

### Determination of cycling genes in tomato

Short reads from a time course experiment performed in 7-day old *S. lycopersicum* var. M82 and *S. pennellii* seedlings were obtained from the SRA database (www.ncbi.nlm.nih.gov/sra, project number PRJNA295848). Although the original experiment consisted in duplicate samples every 4 h during one diel cycle and two circadian cycles, only reads corresponding to the diel cycle were used, corresponding to samples from time-points 12, 16, 20, 24, 28 and 32 (consecutive SRA numbers SRR2452525 to SRR2452536, and SRR2452572 to SRR2452586). Short reads from these 24 libraries were aligned to the tomato genome reference sequence v4.0 using hisat2 v2.1.0 with default parameters [51]. The number of reads per transcript in the ITAG 4.1 annotation was counted with the featureCounts function using the Rsubread R package with default parameters [52]. Low expressed transcripts, that contained less than 10 reads across all samples were discarded. The number of reads in the 24,330 remaining transcripts was normalized using the vst function in the DEseq2 R package [53]. To improve detection of cycling genes, we split the two replicates from each time-point taken in a single day into two consecutive days by adding 24 h to the collection time of the second replicate. Cycling genes were identified independently in *S. lycopersicum* and *S. pennellii* using the meta2d function in the R package MetaCycle with parameters minper = 20, maxper = 28, cycMethod = "ARS", adjustPhase = "predictedPer", combinePvalue = "fisher", ARSmle = "auto" and ARSdefaultPer = 24 [58]. We considered as cycling a total of 5740 genes that presented an adjusted *p* value < 0.05 for the cycling test, an estimated period between 22 and 26 h both in *S. lycopersicum* and *S. pennellii*, and did not present amplitudes or mean expression values more than 2.5 standard deviations away from the mean (Table S3). Since *S. lycopersicum* presents unusually decelerated rhythms [26], cycling genes were assigned the estimated phase from the *S. pennellii* experiment, which was rounded to the nearest integer and subtracted 24 when they exceeded 23 h.

### Molecular timetable studies

For the molecular timetable representation in Fig. 3C, we calculated the Z-score normalized expression of all cycling genes in each genotype, condition and phase bin following the protocol in [59]. For the molecular timetable representation in Fig. 3E, we calculated the average log2 fold expression change due to photoperiod of all cycling genes in each genotype and phase bin. Phases of the curves in Fig. 3C were calculated using the cosinor function in the psych package in R using normalized expression for all cycling genes in each independent biological replicate [60]. Estimation of significant differences in the response to photoperiod for each genotype were calculated using cosinor phases estimates for each independent replicate and a two way ANOVA in the R package emmeans with genotype and condition as factors [61].

## Supplementary Information

**Additional file 1:** **Figure S1.** Whole genome genotyping of a heterozygous line containing reduced introgressions at the positions of LNK2 and EID1. (A) The red line indicates the frequency of heterozygous SNPs in 1000-SNP windows along the 12 tomato chromosomes. (B) Zoom-in view in the chromosomal regions where the heterozygous line presented introgressions from *S. pimpinellifolium*. Red lines indicate the frequency of heterozygous SNPs in 100-SNP windows. The location of LNK2 and EID1 is indicated with a vertical line. The location of genes in each region is indicated with black dots along the x axis. **Figure S2.** Circadian parameters in the near isogenic lines generated. Three independent experiments are shown in columns and Period, Phase, Amplitude and Relative Amplitude Error in rows. The wild species *S. pimpinellifolium* was not included in the first experiment. Different letters in each boxplot indicate significant differences. **Figure S3.** Expression oscillation of LNK2 and EID1 in tomato. Data was obtained from RNA-seq data published in Müller et al 2016. Plants were grown in 12:12 light/dark and 20:18 °C temperature cycles and leaf samples collected from 7-day old seedlings every 4 hours. Read counts on each gene are normalized by gene length and sample size. **Figure S4.** Principal component analysis of expression values from the RNA-seq experiment in the near isogenic lines segregating for wild alleles of EID1 and LNK2. Only transcripts with more than 10 reads across all samples were included in the analysis. **Figure S5.** Phase and percent of differentially expressed genes among cycling genes in tomato. (a) Phase distribution of the 6017 transcripts whose expression oscillates during the diel cycle in *S. lycopersicum* and *S. pennellii.* (b) Percentage of transcripts in (a) whose expression was significantly altered by photoperiod in our experiment. **Figure S6.** Phylogenetic tree from protein sequence alignments for genes belonging to the LNK family in tomato and Arabidopsis. For LNK2, the sequence from the wild tomato species *S. pennellii* is used because of the large deletion present in this gene in cultivated tomato. Arabidopsis, cultivated tomato and *S. pennellii* protein names are highlighted in gray, red and green respectively. Tomato proteins whose transcript oscillates during the diel cycle are marked with an orange dot. 

**Additional file 2:** **Table S1.** primers used to generate near isogenic lines with wild alleles of EID1 and LNK2. **Table S2.** GO enrichment analysis. **Table S3.** List of transcripts whose expression oscillate with ~24h cycles in tomato and their phase estimates.

## Data Availability

The datasets generated and analyzed during the current study are available in the Short Read Archive repository (https://www.ncbi.nlm.nih.gov/sra), under projects PRJNA797239 and PRJNA295848.

## Abbreviations

NILs: Near isogenic lines
MM: *S. lycopersicum* Cv. MoneyMaker
Spim: *S. pimpinellifolium*
LD: Long day photoperiod
SD: Short day photoperiod
ZT: Zeitgeber time
PC: Principal component
RNA-seq: Ribonucleic acid sequencing
ANOVA: Analysis of the variance
QTL: Quantitative trait locus
