# Supplementary material for: Interaction between photoperiod and variation in circadian rhythms in tomato
Source: BMC Plant Biol. 2022 Apr 9;22:187. doi: 10.1186/s12870-022-03565-1 (PMC8994279; doi:10.1186/s12870-022-03565-1)
Supplement: Supplementary file 1 — Additional file 1: Figure S1. Whole genome genotyping of a heterozygous line containing reduced introgressions at the positions of LNK2 and EID1. (A) The red line indicates the frequency of heterozygous SNPs in 1000-SNP windows along the 12 tomato chromosomes. (B) Zoom-in view in the chromosomal regions where the heterozygous line presented introgressions from S. pimpinellifolium. Red lines indicate the frequency of heterozygous SNPs in 100-SNP windows. The location of LNK2 and EID1 is indicated with a vertical line. The location of genes in each region is indicated with black dots along the x axis. Figure S2. Circadian parameters in the near isogenic lines generated. Three independent experiments are shown in columns and Period, Phase, Amplitude and Relative Amplitude Error in rows. The wild species S. pimpinellifolium was not included in the first experiment. Different letters in each boxplot indicate significant differences. Figure S3. Expression oscillation of LNK2 and EID1 in tomato. Data was obtained from RNA-seq data published in Müller et al 2016. Plants were grown in 12:12 light/dark and 20:18 °C temperature cycles and leaf samples collected from 7-day old seedlings every 4 hours. Read counts on each gene are normalized by gene length and sample size. Figure S4. Principal component analysis of expression values from the RNA-seq experiment in the near isogenic lines segregating for wild alleles of EID1 and LNK2. Only transcripts with more than 10 reads across all samples were included in the analysis. Figure S5. Phase and percent of differentially expressed genes among cycling genes in tomato. (a) Phase distribution of the 6017 transcripts whose expression oscillates during the diel cycle in S. lycopersicum and S. pennellii. (b) Percentage of transcripts in (a) whose expression was significantly altered by photoperiod in our experiment. Figure S6. Phylogenetic tree from protein sequence alignments for genes belonging to the LNK family in tomato and Arabidops [file 12870_2022_3565_MOESM1_ESM.docx]

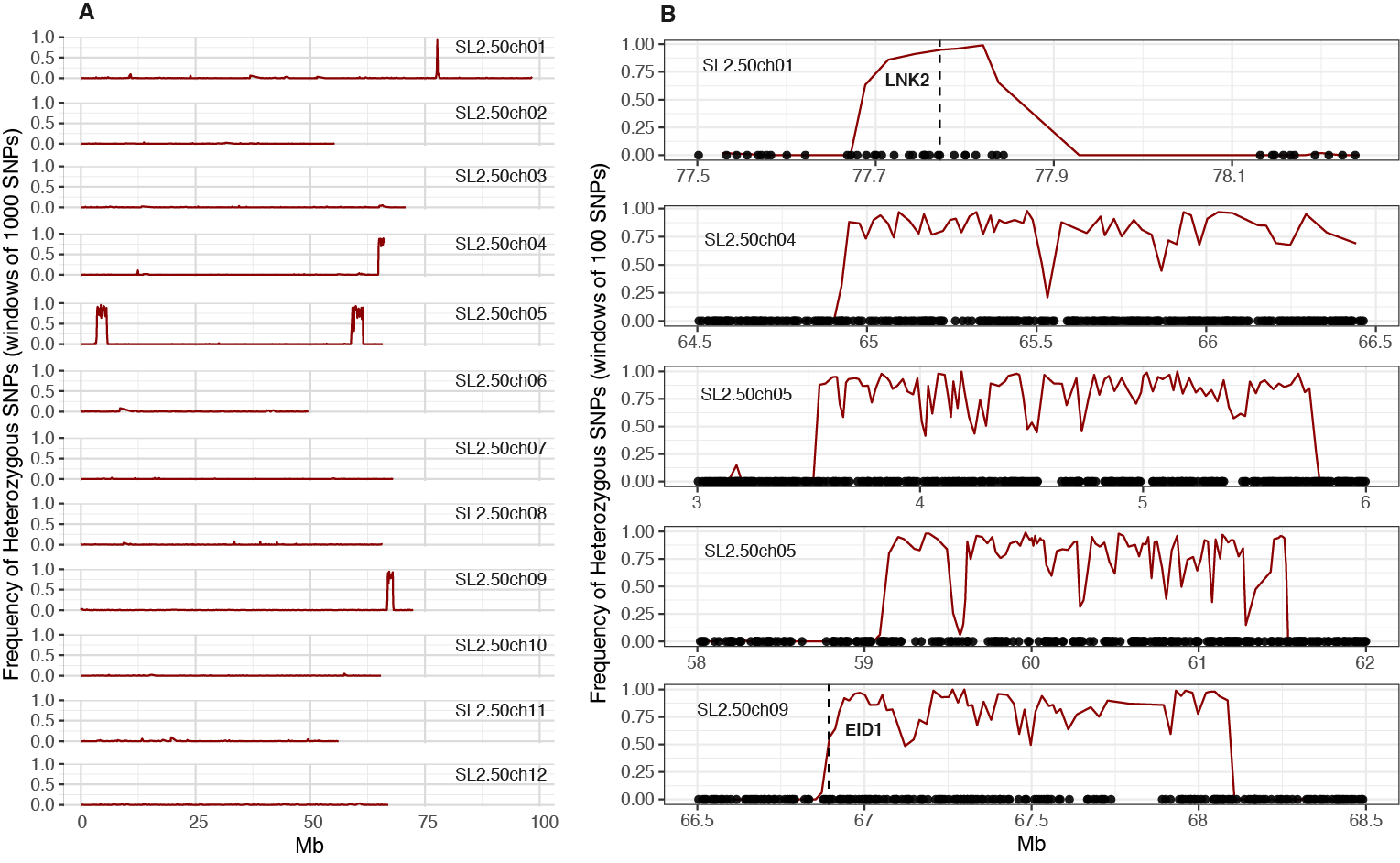


Figure S1. Whole genome genotyping of a heterozygous line containing reduced introgressions at the positions of *LNK2* and *EID1*. (A) The red line indicates the frequency of heterozygous SNPs in 1000-SNP windows along the 12 tomato chromosomes. (B) Zoom-in view in the chromosomal regions where the heterozygous line presented introgressions from *S. pimpinellifolium*. Red lines indicate the frequency of heterozygous SNPs in 100-SNP windows. The location of *LNK2* and *EID1* is indicated with a vertical line. The location of genes in each region is indicated with black dots along the x axis.


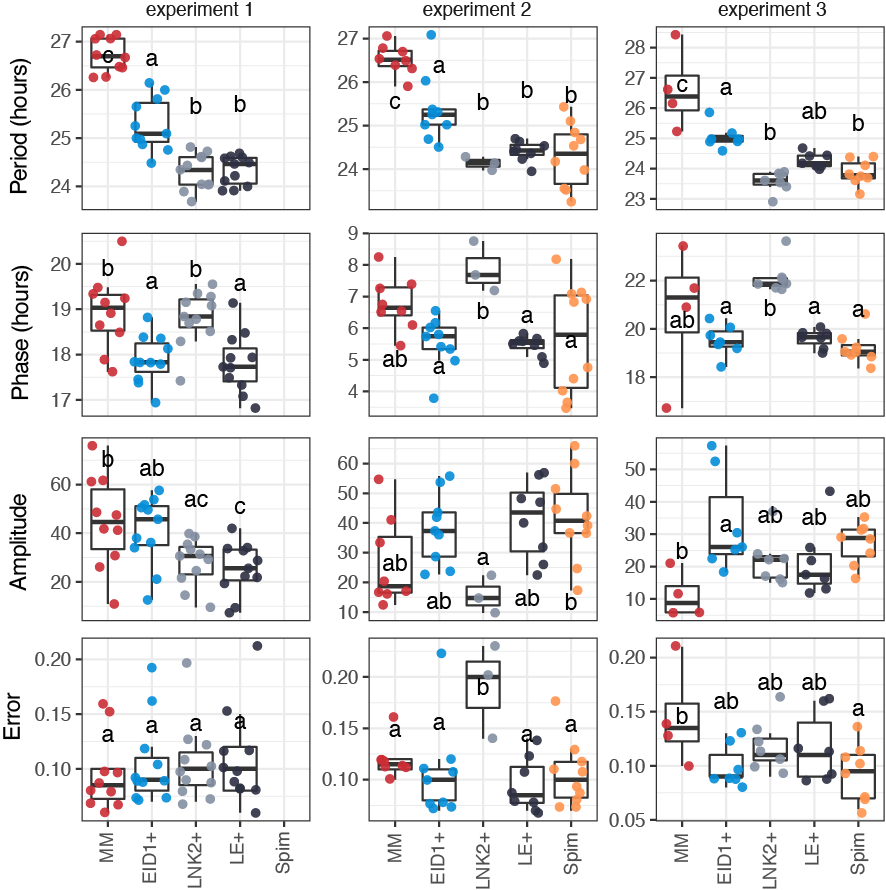


Figure S2. Circadian parameters in the near isogenic lines generated. Three independent experiments are shown in columns and Period, Phase, Amplitude and Relative Amplitude Error in rows. The wild species *S. pimpinellifolium* was not included in the first experiment. Different letters in each boxplot indicate significant differences (P < 0.05, one-way ANOVA and Tukey’s post hoc HSD test).


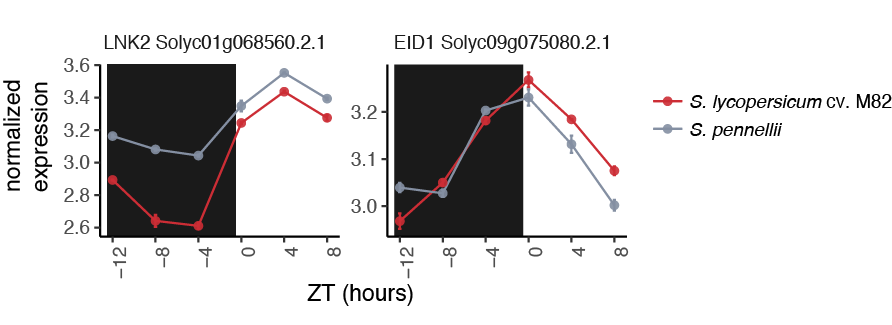


Figure S3. Expression oscillation of *LNK2* and *EID1* in tomato. Data was obtained from RNA-seq data published in Müller et al 2016. Plants were grown in 12:12 light/dark and 20:18 °C temperature cycles and leaf samples collected from 7-day old seedlings every 4 hours. Read counts on each gene are normalized by gene length and sample size.


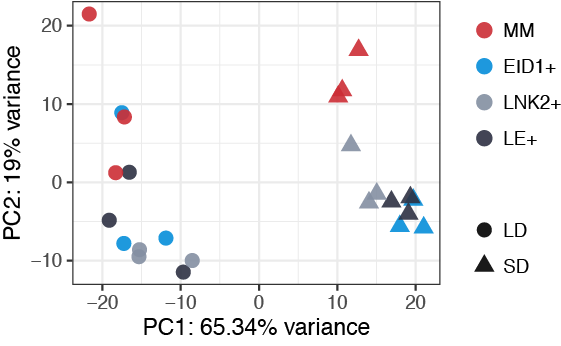


Figure S4. Principal component analysis of expression values from the RNA-seq experiment in the near isogenic lines segregating for wild alleles of *EID1* and *LNK2*. Only transcripts with more than 10 reads across all samples were included in the analysis.


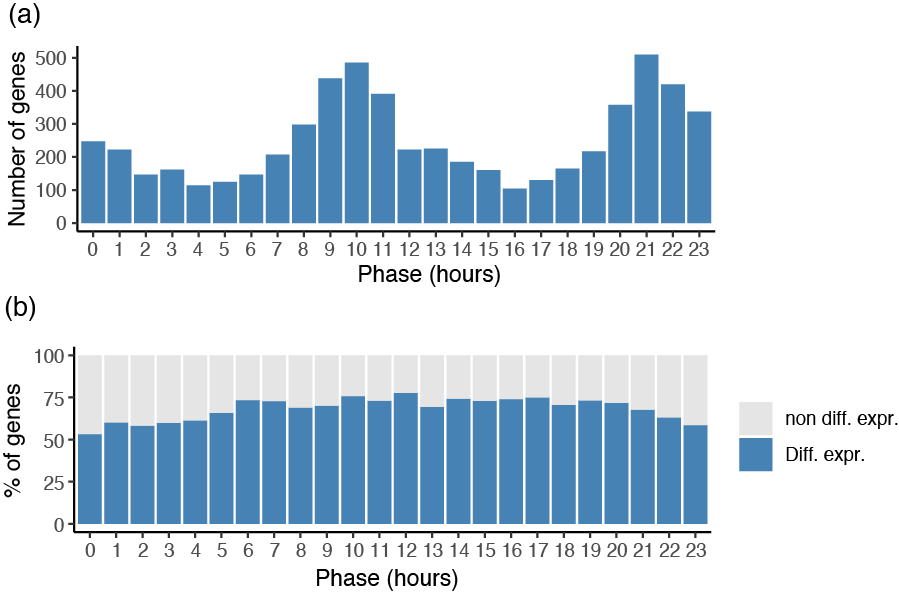


Figure S5. Phase and percent of differentially expressed genes among cycling genes in tomato. (a) Phase distribution of the 6017 transcripts whose expression oscillates during the diel cycle in *S. lycopersicum* and *S. pennellii*. (b) Percentage of transcripts in (a) whose expression was significantly altered by photoperiod in our experiment.


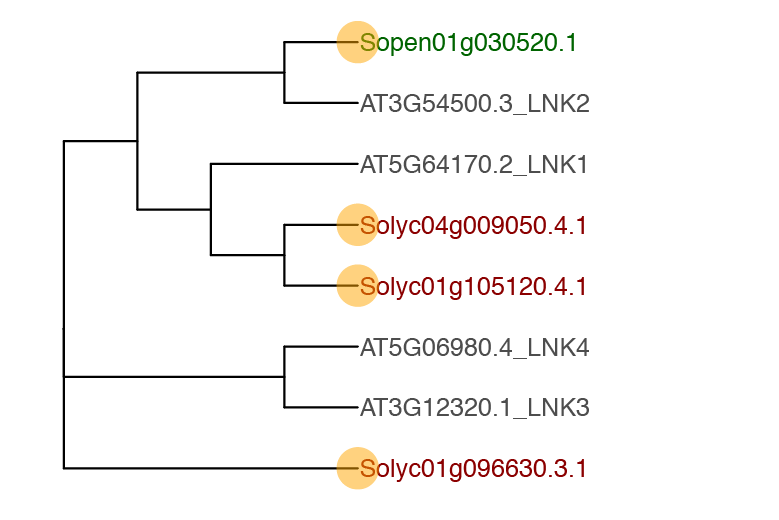


Figure S6. Phylogenetic tree from protein sequence alignments for genes belonging to the LNK family in tomato and Arabidopsis. For *LNK2*, the sequence from the wild tomato species *S. pennellii* is used because of the large deletion present in this gene in cultivated tomato. Arabidopsis, cultivated tomato and *S. pennellii* protein names are highlighted in gray, red and green respectively. Tomato proteins whose transcript oscillates during the diel cycle are marked with an orange dot.
